# Modulatory Effects of Attention on Lateral Inhibition in the Human Auditory Cortex

**DOI:** 10.1371/journal.pone.0149933

**Published:** 2016-02-22

**Authors:** Alva Engell, Markus Junghöfer, Alwina Stein, Pia Lau, Robert Wunderlich, Andreas Wollbrink, Christo Pantev

**Affiliations:** 1 Institute for Biomagnetism and Biosignalanalysis, University Hospital Muenster, Muenster, Germany; 2 Institute for Medical Psychology and Systems Neuroscience, University of Muenster, Muenster, Germany; 3 Institute for Physiological Psychology, University of Bielefeld, Bielefeld, Germany; University of Salamanca- Institute for Neuroscience of Castille and Leon and Medical School, SPAIN

## Abstract

Reduced neural processing of a tone is observed when it is presented after a sound whose spectral range closely frames the frequency of the tone. This observation might be explained by the mechanism of lateral inhibition (LI) due to inhibitory interneurons in the auditory system. So far, several characteristics of bottom up influences on LI have been identified, while the influence of top-down processes such as directed attention on LI has not been investigated. Hence, the study at hand aims at investigating the modulatory effects of focused attention on LI in the human auditory cortex. In the magnetoencephalograph, we present two types of masking sounds (white noise vs. withe noise passing through a notch filter centered at a specific frequency), followed by a test tone with a frequency corresponding to the center-frequency of the notch filter. Simultaneously, subjects were presented with visual input on a screen. To modulate the focus of attention, subjects were instructed to concentrate either on the auditory input or the visual stimuli. More specific, on one half of the trials, subjects were instructed to detect small deviations in loudness in the masking sounds while on the other half of the trials subjects were asked to detect target stimuli on the screen. The results revealed a reduction in neural activation due to LI, which was larger during auditory compared to visual focused attention. Attentional modulations of LI were observed in two post-N1m time intervals. These findings underline the robustness of reduced neural activation due to LI in the auditory cortex and point towards the important role of attention on the modulation of this mechanism in more evaluative processing stages.

## Introduction

Perception and processing of an auditory stimulus strongly depends on the properties of preceding sounds. One example is the reduced neural activation elicited by a tone when preceded by a sound whose spectral range closely frames the tone’s frequency. This observation has been demonstrated by means of neural correlates of auditory processing like the N1m and is explained by the mechanism of lateral inhibition (LI) in the auditory system[1–4]. It has been argued, that LI occurs as a result of the processing of auditory inputs by inhibitory interneurons in the auditory cortices[5–7]. Additionally to excitatory pathways these inhibitory interneurons can be found in the auditory cortices and are assumed to inhibit lateral neurons with higher and lower characteristic frequencies [8]. These interneurons play an important role in the auditory processing as they for example enhance spectral energy contrasts in auditory stimuli [9]. When the auditory system is presented with a stimulus with high spectral energy contrasts, e.g., a white noise where a specific frequency range is removed by a notch filter, neurons coding the frequencies within the frequency range of the notch do not get activated and therefore do not induce inhibition on neighbouring neurons. Neurons coding the edge frequencies around the notch-filtered region become more excited and spread increased inhibition onto neighbouring neurons, especially those which code the notch-filtered frequencies. Consequently, the processing of a following tone with a frequency corresponding to the centre of the notch-filter is reduced, as demonstrated by a significant reduction of the auditory evoked N1m response [1–4]. This mechanism of LI is not only important for basic auditory perception, but might also play a major role in the assumed generation and possible treatment of symptoms like tinnitus, i.e., the sustained perception of sound without an external source (e.g., [10]). For instance, one year of listening to notch-filtered music, that induced LI on the neurons coding the frequencies corresponding to the frequency of the tinnitus tone, decreased the subjectively perceived loudness of the tinnitus [11]. For a detailed overview on the suggested impact of LI on generation and treatment of tinnitus see [12,13].

So far, studies which investigated LI in the human auditory cortex focused on examining effects of certain stimulus characteristics like notch width, localization of the notch, time interval between the masking sound and the test tone (TT) and the effect of edge frequency bandwidth on the N1m reduction due to LI [2–4,14]. In contrast to this variety of studies examining the influence of bottom-up effects on LI, there are to our knowledge no studies directly addressing the question of top-down influences, such as focused attention, on LI effects. In previous studies investigating LI in the human auditory cortex, the focus of attention was typically directed away from the auditory modality as subjects watched silent movies during the presentation of the auditory stimuli [2–4]. As the auditory masking sounds and test tones were repeated many times, it can be assumed, that these were perceived rather inattentively as stimulus without relevance similar to background noise. The fact that frequency specific N1m suppression after notched noise was found in these studies, clearly indicates that LI can occur during passive listening in complete absence of directed attention. Hence, focused attention does not seem to be a necessary prerequisite for LI. However, several studies on the effect of directed attention on auditory processing point towards the hypothesis, that a focus on the auditory modality might enhance the effect of LI (e.g., [15–18]). The effect of LI is usually examined by means of the amplitude reduction of the N1m [1–4]. This sensory component is known to be influenced not only by bottom-up but also by top-down processes [18,19] and many studies revealed that the magnitude of the N1m is modulated by directed attention (e.g., [15–18]). Under focused attention towards the stimulus, the N1m has been shown to be larger than under distracted attention. These effects could be demonstrated for unimodal paradigms such as dichotic listening [15,17] as well as for multimodal attentional paradigms in which auditory stimuli had to be evaluated, ignored or a distraction task referring to the visual modality was performed [3,20,21]. The latter paradigms are especially suited to make inferences about the neural processes underlying auditory attention, as they do not evoke conflicting interactions between left and right hemispheric brain activation during binaural compared to monaural listening [20,22]. In recent studies, the observation of an N1m increase due to focused attention was explained in terms of a combination of increased neural activity level (gain model; [17,23]) and an enhanced frequency selectivity in auditory cortices due to selective attention (tuning/sharpening model [20,24–26]). Considering the mechanism of LI, a gain increase together with a sharper tuning of auditory neurons could in fact enhance the amount of inhibition which is spread to the neurons coding the center frequency of the notch, leading to a larger reduction of neural activity due to LI under focused attention. First hints towards such a mechanism have recently been shown by Pape and coworkers [27], who examined the prolonged influences of LI due to notch-filtered music, namely “inhibition-induced plasticity” [28]. They demonstrated that in tinnitus patients inhibition-induced plasticity only emerged under focused attention on the music and it was not observable in a condition were subjects actively produced the notch-filtered music. This latter condition can be seen as a form of divided attention, as subjects had to split their attentional resources between the two tasks “producing” the music and “listening” to it. Hence, these results point towards smaller effect of LI under divided attention. Albeit interesting, these results need to be investigated in more detail, as the study assessed only a small population of subjects suffering from tinnitus and the study design was too complex to derive distinctive evidence about the influence of attention on LI in the human auditory cortex.

Taken together, we hypothesize that the effect of LI in the auditory cortices should be significantly larger when attention is directed towards the auditory modality (focused attention) as compared to the condition, where attention is directed towards the visual and thus away from the auditory modality (distracted attention). Besides this main hypothesis, we expect to replicate previous findings of a reduced N1m for a tone following notched white noise as compared to the N1m for a tone following white noise without a notch. As previous research on LI focused on the effect of LI in the auditory cortices, we formulated our hypotheses with respect to this brain region, only. To test these hypotheses, we chose stimuli which had been shown to evoke the strongest LI effects in a passive viewing paradigm with sequential masking [2] and included two tasks to modulate the focus of attention, adapted from a study by Stracke and colleagues [21]).

## Methods

### Subjects

Twenty-seven subjects with normal hearing participated in the experiment (HL < 20 dB). The study was conducted according to the Declaration of Helsinki and the study protocol was approved by the ethics committee of the medical faculty of the University of Muenster. Subjects received written and oral information about the procedure of the study and signed an informed consent form.

### Experimental design and stimulation

**Fig 1:**
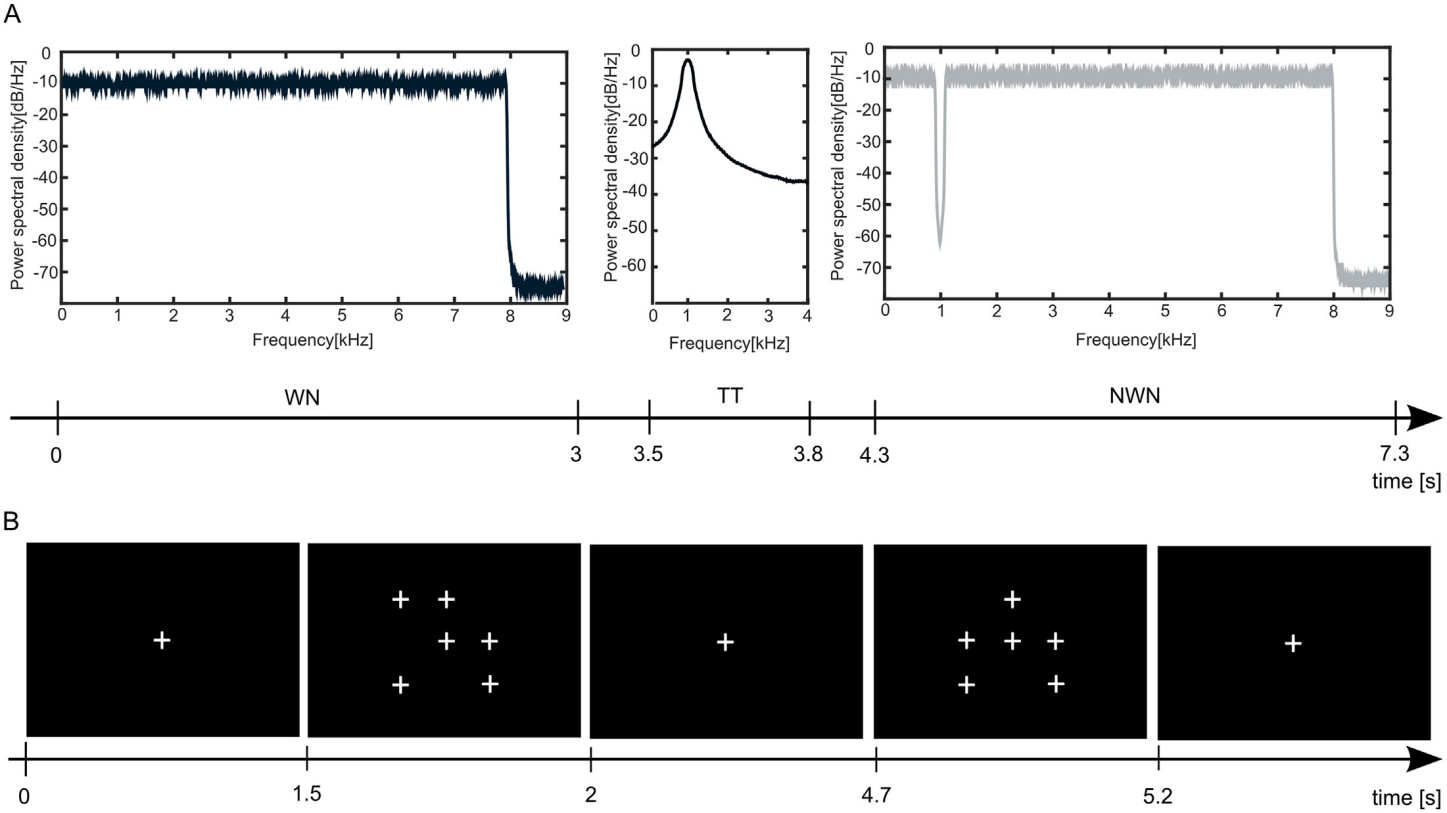
Overview of the stimulation paradigm. Subjects were presented with visual and auditory stimuli simultaneously. See text for a detailed description of the stimulation. (A) Auditory stimulation, comprising a white noise (WN) masking stimulus, a test tone (TT) and a notched white noise (NWN) masking stimulus. (B) Visual stimulation, consisting of patterns of small white crosses presented on the screen. As the duration for the presentation of the fixation cross (first stimulus) was randomly jittered between 0.2 and 3 s, the displayed timing of the stimuli represents only one possible example. On one half of the trials, subjects were instructed to concentrate on the auditory input to detect small changes of loudness in the sounds. On the other half of the trials subjects were instructed to concentrate on the visual input and detect target stimuli on the screen.

**Fig 2:**
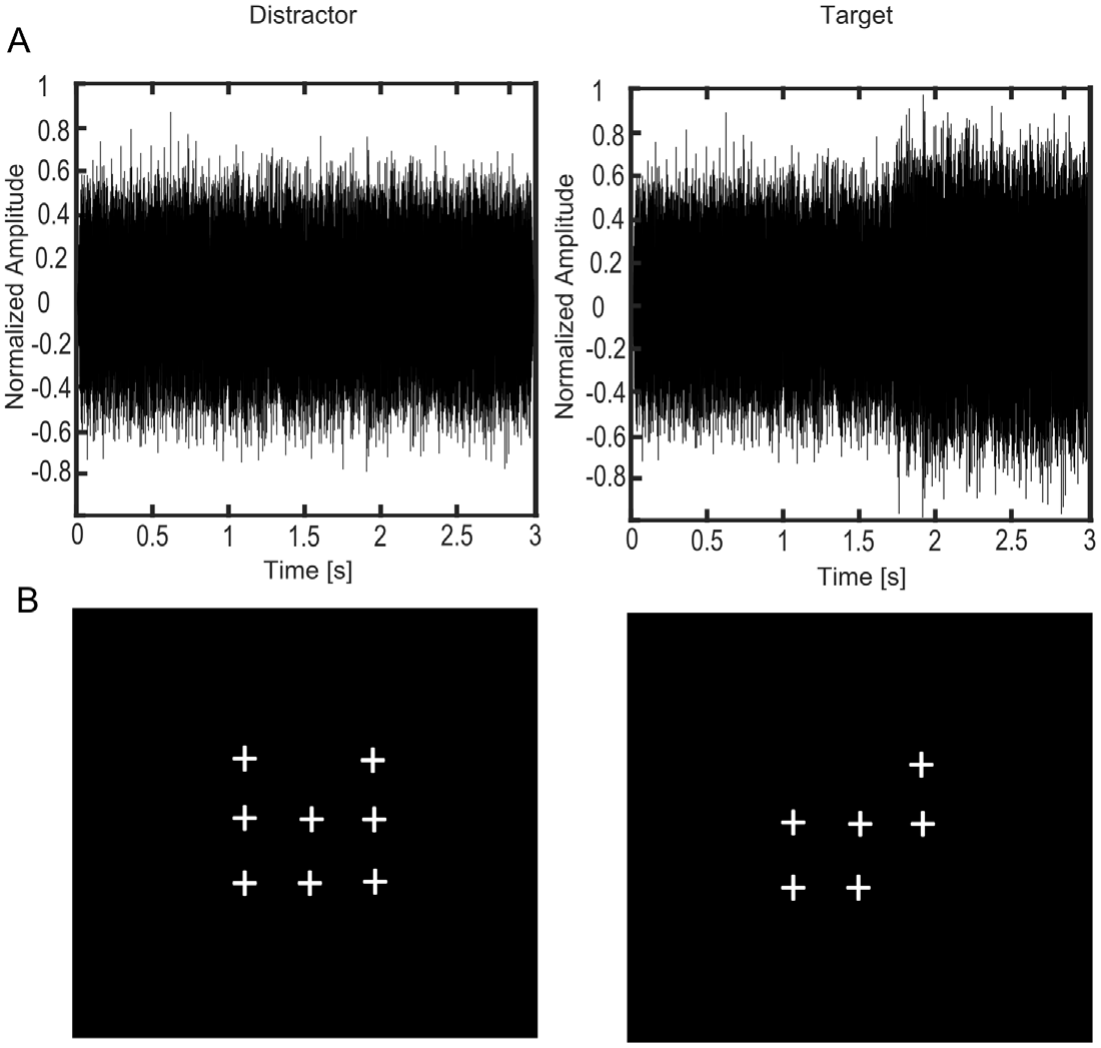
Depiction of target and distractor stimuli for the visual and auditory tasks. (A) Auditory distractor and target: Small changes of loudness (increase of 2 dB) were implemented as the target masking sound. This loudness change could occur on four different time points during the presentation of the masking stimulus (0.5 s, 1.1 s, 1.7 s, 2.6 s) and lasted until the end of the masking stimulus. The depiction shows a loudness change at 1.7 s. (B) Visual distractor and target stimuli: When the pattern of small crosses displayed exactly one small square of crosses, the stimuli were considered as targets.

Auditory and visual stimuli were presented simultaneously and independently from each other in four runs during magnetoencephalographic measurements. Before the first measurement in the magnetoencephalograph (MEG), a standardized procedure was run to determine the individual hearing threshold for the auditory stimuli presented by special earphones in the magnetically shielded room. Please see Fig 1 for an illustration of the stimulation paradigm. The auditory stimulation comprised two types of masking stimuli and one test tone (TT) which consisted of a 1000 Hz pure tone (spectrum at Fig 1A center). As masking stimuli we used white noise (WN, spectrum at Fig 1A left) or white noise passing through a digital notch filter centered at 1 kHz (notch depth 40 dB, bandwidth ¼ octave) further referred to as notched white noise (NWN, spectrum at Fig 1A right). In 10% of the masking stimuli, a small change of loudness (increase of 2 dB) was implemented as the target masking sound (cf. Fig 2A). This loudness change could occur on four different time points during the presentation of the masking stimulus (0.5 s, 1.1 s, 1.7 s, 2.6 s) and lasted until the end of the masking stimulus. The total root-mean-square (RMS) values for all masking stimuli were counterbalanced at the same level. All stimuli had a 0.02 s rise and fall time and were low-pass filtered at 8 kHz adjusted to the frequency characteristics of the sound delivery system. The auditory stimuli were presented binaurally with an intensity of 45 dB SL (sensation level, corresponding to the individual hearing threshold for the TT).

Each trial consisted of a 3 s masking sound followed by the TT for 0.3 s with an inter stimulus interval of 0.5 s. The different masking and target masking sounds were presented in randomized order. Stimuli were presented in four runs with a short break in between. Each run consisted of 70 trials per masking condition and 14 trials with a target masking sound. This added up to 140 trials per run, i.e., 560 trials in total. The different target masking sounds were randomized across runs.

The visual stimuli consisted of small crosses (“+”) presented on the screen at one to nine predefined positions (for a similar task cf. [21,29]). The cross at the centre of the screen served as a fixation point and was present during the entire measurement. The presentation time for a given pattern of the crosses was 0.5 s. Between the presentation of the patterns, the cross at the centre of the screen remained visible without any other crosses for a time interval jittered between 0.5 and 3 s. In 10% of the trials crosses appearing on the screen formed a single small square (visual targets, cf. Fig 2B). 140 visual patterns were presented in one run.

To manipulate the focus of attention, subjects had to perform two different tasks during the MEG measurements. Please see Fig 2 for a depiction of auditory and visual distractor and target stimuli. In two of the four runs, subjects were instructed to concentrate on the auditory modality and press a button as quickly and accurately as possible with their right index finger, if they detected a loudness change in the masking sounds (focused auditory attention). In the other two runs, subjects were instructed to direct their attention to the visual domain. In this task, they had to press a button as quickly and as accurately as possible when they detected exactly four neighbouring crosses arranged in a small square in the visual pattern (distracted attention). During all runs, we instructed the subjects to fixate the cross at the centre of the screen and avoid eye movements.

### Data acquisition

MEG recordings were performed in a magnetically shielded room, using a 275-sensors whole-head MEG system (Omega 275, CTF, VSM MedTech Ltd.) with first-order axial SQUID gradiometers (2 cm diameter, 5 cm baseline, 2.2 cm average inter-sensor spacing) The acquisition of the data took place between May and August 2015. Data was recorded continuously with a sampling rate of 600 Hz in the frequency band between 0 and 150 Hz. Subjects were sitting upright in the MEG with their head stabilized by cotton patches inside the dewar to prevent head movements. The position of the head in the MEG scanner was recorded by fiducials which were affixed inside the ears and upon the nasion. Behavioural data was collected by custom made opto-electric response buttons.

### Data analysis

Only neural responses evoked by the TT were considered for the analysis. Data were off-line filtered between 0.1 Hz and 30 Hz. The averaging epoch was defined from 300 ms before to 500 ms after TT onset and the data was baseline-corrected using a 100 ms pre-stimulus interval. Trials comprising target masking sounds or button presses within a time interval of 1 s before and 1 s after TT onset were excluded from averaging. MEG data filtering and distinction of the epochs was done with standard programs belonging to the analysis software package provided by the MEG system manufacturer and several custom made programs adapted from those. For artefact detection, averaging, inverse modelling, statistical analysis and visualization of results we used the Matlab-based EMEGS software (www.emegs.org; [30]). Artefact detection and rejection was performed with an established method for statistical control of artefacts in high- density EEG/MEG data [31]. This procedure (1) detects individual channel artefacts; (2) detects global artefacts; (3) replaces artefact-contaminated sensors with spline interpolation, statistically weighted on the basis of all remaining sensors; and (4) computes the variance of the signal across trials to document the stability of the averaged waveform. The rejection of artefact-contaminated trials and sensor epochs relies on the calculation of statistical parameters for the absolute measured magnetic field amplitudes over time, their standard deviation over time, as well as on the determination of boundaries for each parameter. Here we decided to reject ocular related artefacts and not try to correct them. Ocular artefact corrections have the advantage of rescuing trials which may enhance the signal to noise ratio, but, in case of slightly incorrect correction, might result in a spatial projection of artefacts into non-ocular regions, for instance the auditory sensory target regions. Residual artefacts of slightly incorrect rejections on the other hand would, at least in a distributed source model as used here, result in artificial activity within the eye-regions and would not affect the estimated neural activity within auditory sensory target regions.

After averaging, the underlying neural network activity was estimated by means of the least square minimum-norm estimation method (L2-MNE, [32]). We chose a distributed source model to disentangle the multiple neural sources that might be involved in the processing of the TT in different attentional states. This procedure enabled us to focus our analysis on activity solely evoked in the auditory cortices, without influence of neural activity originally evoked in different brain regions. As head model, a spherical shell with 8 cm radius was used. On this spherical shell 350 tangentially oriented test dipoles were evenly distributed. Information about the positions of the MEG sensors relative to the head was used to calculate individual lead-field matrices for each participant (Tikhonov regularization parameter λ = .1). These calculations resulted in source waveforms over time for each test dipole.

**Fig 3:**
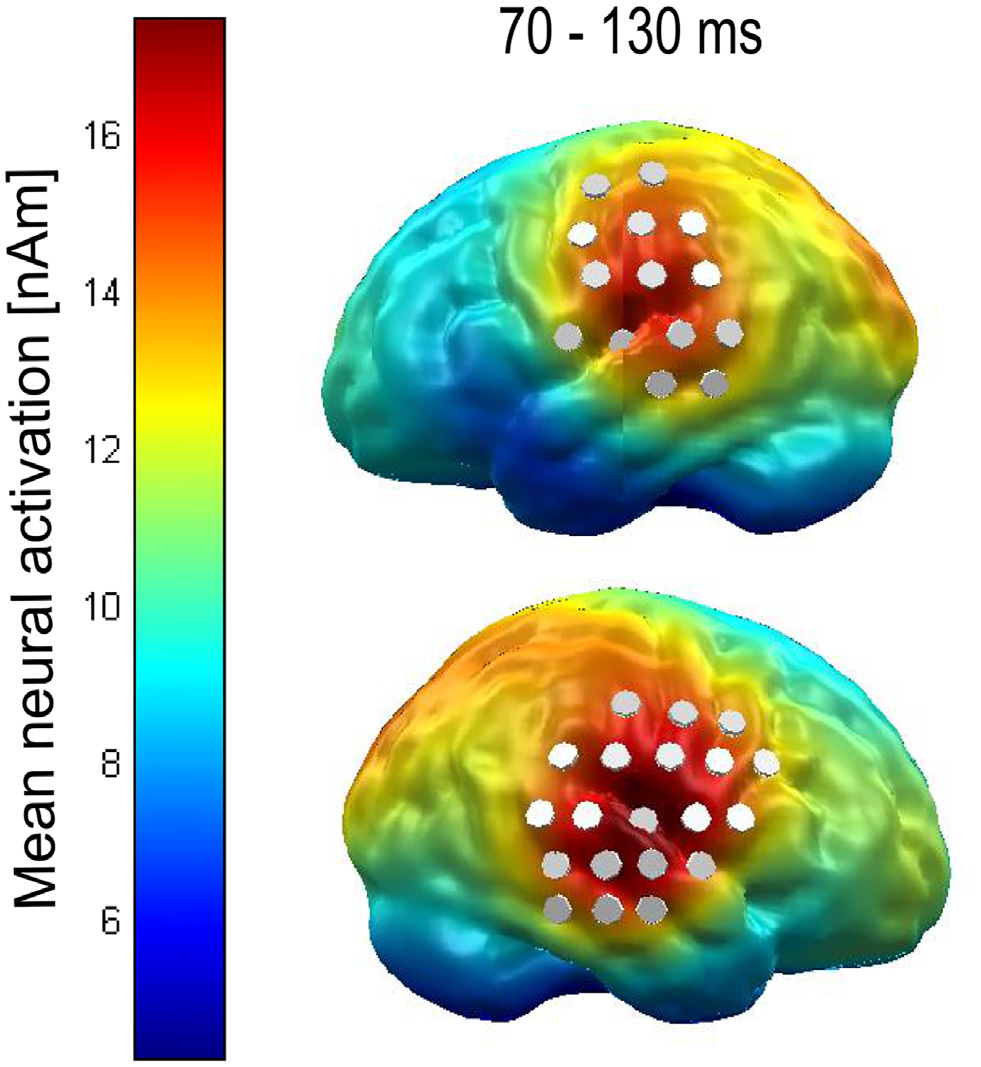
Depiction of the dipoles chosen as the auditory region of interest (ROI). Mean neural activation for the TT over all conditions was averaged between 70 and 130 ms and plotted on a cortical surface. Grey cylinders indicate the locations of selected dipoles.

The L2-MNE values were entered in a repeated measures Analysis of Variance (ANOVA) over all time points and dipoles with the within subjects factors *noise type* (white noise vs. notched white noise) and *attentional focus* (auditory vs. visual). The statistical analyses were corrected for multiple comparisons using cluster-based permutation tests [33]. Based on a-priori hypotheses on location (auditory cortices) and latency (N1m time window) of the expected LI effects, we integrated the following spatio-temporal priors in the multiple testing procedure: Analogue to previous literature on the timing of the effect of LI in the auditory cortex [2,4], we used the temporal prior of a time window between 70 and 130 ms for the cluster-based permutation test. To define the spatial prior, i.e., a bilateral region of interest (ROI) encompassing the area of the auditory cortices, we averaged the neural activation evoked by the TT across all conditions and runs (i.e., the main effect of the TT) and plotted the result on a cortical surface for the time interval between 70–130 ms. Within this time interval we defined those dipoles as ROI which showed the strongest activation in the left and right hemisphere (cut off: 80% of maximal neural activity, cf. Fig 3). This bilateral ROI and the assumption of hemispherical symmetry were then introduced as spatial priors into the cluster based permutation test. The Monte Carlo *p*- values were set to *p* < .05 on sensor and *p* < .05 on cluster level and 1000 permutations were drawn.

In an additional analysis, we conducted another cluster-based permutation test without temporal priors (0–500 ms). This analysis was done to investigate the effect of LI outside the N1m time window (analogue to [2,3]) and to examine putative effects of attention on auditory processing that might occur in later latency intervals [34]. To render this additional analysis more conservative, the Monte Carlo *p*-value was set to *p* < .05 on sensor and *p* < .01 on cluster level.

In case of significant interactions, we used paired *t*-tests to disentangle the interaction effects. Here, we first calculated the difference between the neural activation evoked by the TT when preceded by WN and the neural activation evoked by the TT when preceded by NWN (TT_WN_-TT_NWN_) for each of the two attentional conditions separately. These difference values, further referred to as “LI-effect” were then introduced into the paired *t*-test to compare them between the two attentional conditions. As the hypotheses were formulated as one-sided, the respective *p*- values are reported as such.

For the statistical analysis of the behavioural data, *d*-prime values for both the visual and the auditory task were calculated using the formula *Z*(Hitrate)–*Z*(False-alarmrate) [35]. The resulting *d*-prime values were then introduced into a paired *t*-test to compare the task difficulty between the visual and auditory task.

## Results

**Fig 4:**
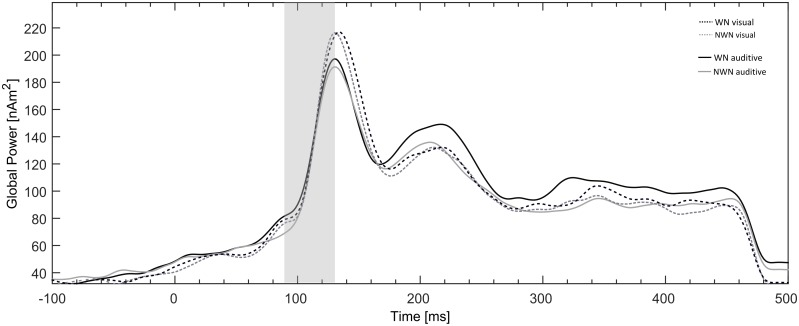
Global source power plot of the source waveforms averaged across all diploes and all subjects. Neural activities evoked by the TT following white noise (WN, black) or notched white noise (NWN, grey) during the visual task (dotted line) and the auditory task (solid line). The a priori defined N1m time interval (70–130ms) of interest is highlighted in grey.

We analysed the data of 21 subjects (M = 27.24 years, SD = 5.51, 13 females). Data of six subjects were excluded from analysis as they showed continuous eye movements during the measurement especially in the auditory runs. Hence, a large amount of trials had to be rejected in the artefact scan of these subjects, leading to insufficient signal-to noise ratio for further analyses. We assume that these six subjects did not follow the instruction to keep their eyes focused on the fixation cross during all runs of MEG measurement. Fig 4 shows the global power over all dipoles averaged over the 21 subjects in the time window between -100 and 500 ms around the onset of the TT and the a-priori chosen N1m time interval of interest (70–130 ms).

### Effect of LI

**Fig 5:**
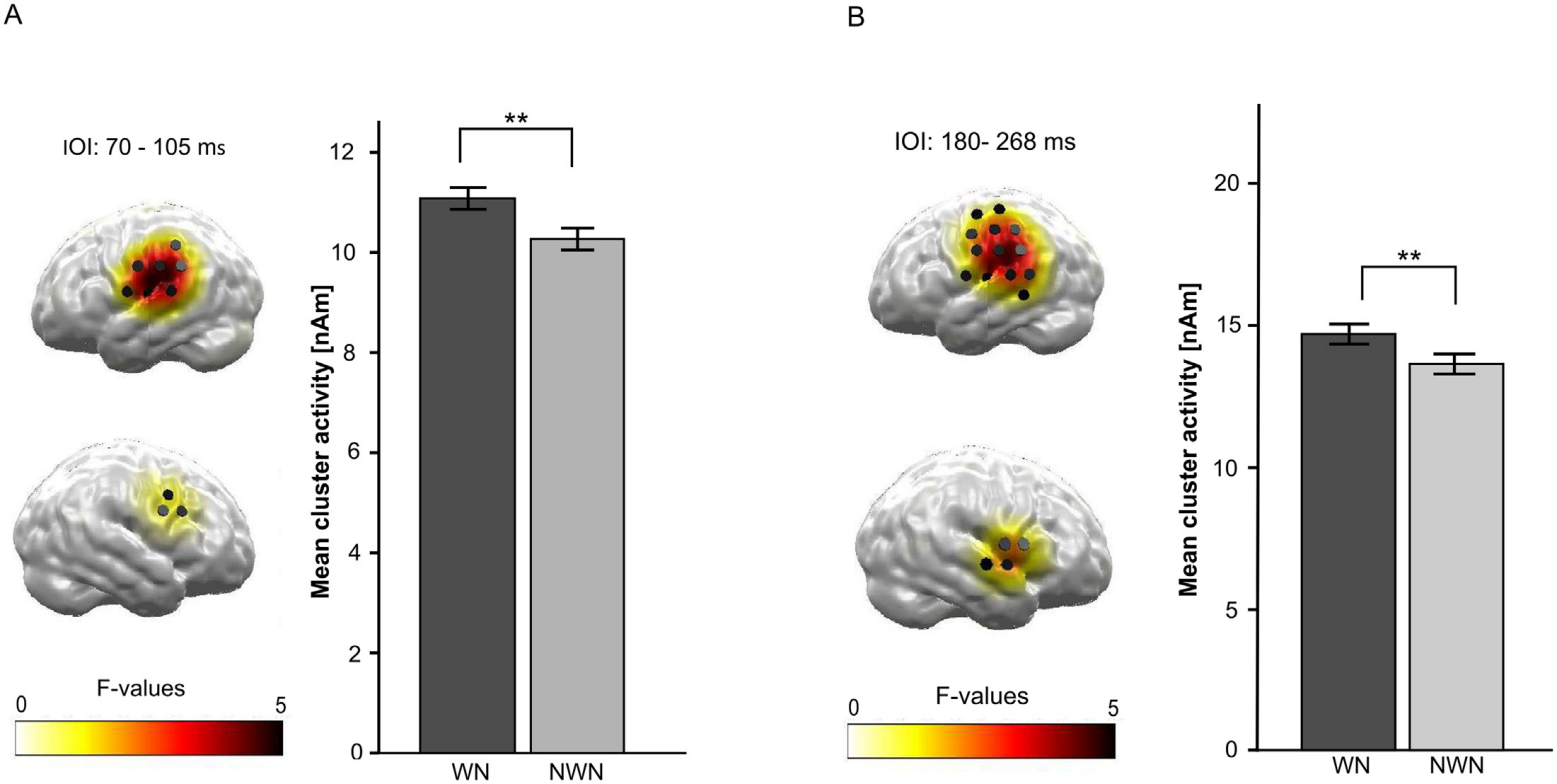
Results for the main effect of *noise type* in the bilateral temporal region of interest (ROI) for the two different intervals of interests (IOI). Only regions surviving cluster-based permutation are colorized. *F*-values corresponding to a corrected *p* < .05(A) or *p* < .01. (B) Black cylinders indicate the locations of dipoles selected as the temporal ROI.** *p* < .01. (A) Left: Statistical parametric map of *F*-values for the main effect *noise type* averaged between 70 and 105 ms. Right: Corresponding mean ROI activity for the test tone (TT) after white noise (WN, black) and after notched white noise (NWN, grey). Bars denote within-subject 95%-confidence intervals. (B) Left: Statistical parametric map of *F*-values for the main effect *noise type* averaged between 180 and 268 ms. Right: Corresponding mean ROI activity evoked by the TT after WN (back) and after NWN (grey). Bars denote within-subject 95%-confidence intervals.

In the analysis with spatio-temporal priors (bilateral auditory cortices, 70–130 ms) a bilateral significant cluster was found with a temporal extension from 70 to 105 ms, *F*(1, 20) = 15.701, *p* = .001, *η*_*p*_*²* = .440. This effect showed a decrease in activation for the TT following the NWN masker compared to the TT following the WN masker (Fig 5A). When considering the whole time interval, one additional cluster in a later time interval was found ranking from 180 ms to 268 ms, *F*(1, 20) = 10.279, *p* = 0.002 *η*_*p*_² = .339. This effect mirrored the effect found in the N1m time interval (Fig 5B).

### Interaction between LI and attention

**Fig 6:**
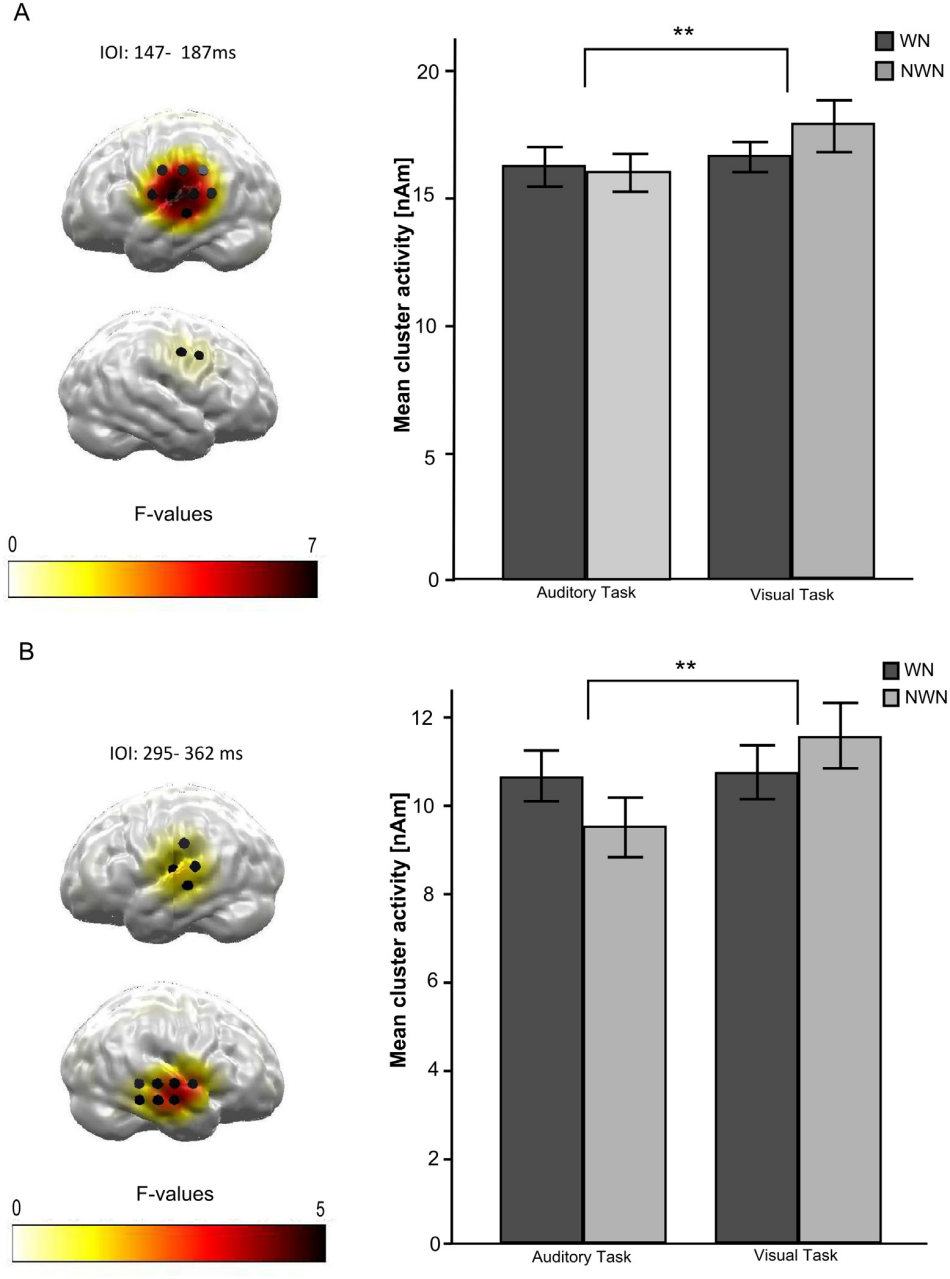
Results for the interaction effect of *noise type x attentional focus* in the bilateral temporal region of interest (ROI) for the two different intervals of interests (IOI). Only regions surviving cluster-based permutation are colorized. *F*-values corresponding to a corrected p < .01. Black cylinders indicate the locations of dipoles selected as the temporal ROI. **p < .01. (A) Left: Statistical parametric map of *F*-values for the interaction effect *noise type x attentional focus* averaged between 147 and 187 ms. Right: Corresponding mean ROI activity evoked by the TT after WN (black) and after NWN (grey) presented separately for the auditory and visual task. Bars denote within-subject 95%-confidence intervals (B) Left: Statistical Parametric Map of *F*-values for the interaction effect *noise type x attentional focus* averaged between 295 ms and 362 ms. Right: Corresponding mean ROI activity evoked by the TT after WN (black) and after NWN (grey) presented separately for the auditory and visual task. Bars denote within-subject 95%-confidence intervals.

In the cluster based permutation test with spatio-temporal priors for the interaction effect *noise type x attentional focus* no significant cluster remained. However, when the whole time interval between 0 and 500 ms was analyzed, two significant clusters were found (Fig 6): One cluster between 147 and 187 ms, *F*(1, 20) = 8.207, *p* = 0.005, *η*_*p*_*²* = .291 and a second cluster between 295 and 362 ms, *F* (1, 20) = 10.739, *p* = 0.002 *η*_*p*_*²* = .349. Within the significant clusters post hoc *t*-tests were calculated to compare the magnitude of the LI effect (TT_WN_-TT_NWN)_ between the two attentional conditions. For both bilateral clusters, results indicate a significantly larger difference between the neural activation for the TT following WN compared to the TT following NWN during auditory attention compared to the respective difference (TT_WN_-TT_NWN_) during visual attention, first cluster: *t*(20) = 2.867, *p* = .005; second cluster: *t*(20) = 3.457, *p* = .001.

### Behavioral results

An overview of the behavioral data can be found in Table 1. A paired t-test on the *d*-prime values in the two attentional conditions showed significantly larger values in the visual compared to the auditory task *t*(20) = -2.23, *p* = .037.

**Table 1:** Behavioural data for the auditory and the visual task separately.

	Visual task Mean (SD)	Auditory task Mean (SD)
**D-Prime**	4.551 (0.851)	3.914 (1.071)
**Hit rate**	.978 (.026)	.882 (.088)
**False-alarm rate**	.020 (.016)	.020 (.053)

### Additional analysis

As the behavioral results indicate a significant difference in task difficulty an analysis of covariance (ANCOVA) was performed to control for the putative effect of task difficulty on the interaction effect between LI and attention. To perform this analysis, we first calculated the difference between *d*-prime values in the visual and auditory task for each subject (*d*-prime visual–*d*-prime auditory). These difference scores were then introduced as covariate in an ANCOVA with the factors *noise type* and *attentional focus*. This ANCOVA was performed on the mean neural activity in the two clusters which had shown a significant *noise type x attentional focus* interaction. Results revealed no significant influence of the difference in the *d*-prime values on the interaction between *noise type* and *attentional focus* (both *p* >.5) and both interaction effects remained significant even when controlling for the effect of task difficulty (first cluster between 147 and 187 ms: *F*(1, 20) = 4.970, *p* = 0.038, *η*_*p*_*²* = .207; second cluster between 295 and 362 ms: *F* (1, 20) = 6.291, *p* = 0.021 *η*_*p*_*²* = .249).

## Discussion

This study aimed at investigating the effect of focused attention on the magnitude of LI in the human auditory cortex. To isolate the influence of focused attention on LI, sensory input was kept identical between the two attentive conditions.

In the analysis of a bilateral ROI in temporal regions, the effect of an N1m reduction due to LI was found in the data, as the TT evoked less neural activity when presented after the NWN masker compared to the WN masker condition. Hence, the finding of an N1m reduction due to LI was replicated and extended by the use of a distributed source model reconstructing sensory neural activity independent of potential simultaneous activity in other brain regions. It was shown, that analogue to the findings in an equivalent current dipole (ECD) model [1,3,4], in a distributed source model the effect of LI could be demonstrated in a region encompassing the auditory cortices. This effect was present in the time window of the N1m (70–105 ms) as well as in a later time window between 180 and 268 ms. The timing of the later LI effect fits the time window of the P2 component (around 160–200 ms) which is assumed to reflect classification and evaluation of the auditory stimuli [36,37]. Hence, the later effect points to the assumption, that the effect of LI persists longer than demonstrated in previous studies and affects later and more evaluative processing stages in audition as well. Previous studies on LI applying the ECD model did not demonstrate an effect of LI in the P2 time window [2] or were not able to analyze the effect of LI on the P2m, due to insufficient to signal-to-noise-ratios [3]. However, in a study focusing on inhibition-induced plasticity due to the induction of LI in the auditory cortex of tinnitus patients, where a distributed source model was applied, an inhibition-induced plasticity was found in a post N1m time interval comparable to the study at hand (150 to 250 ms cf. [28]). Hence, it could be hypothesized, that the distributed source model might be better suited to investigate the effect of LI in both N1m and later time windows when significant activity variance occurs simultaneously at other cortical regions. Our finding might be interpreted as a hint for the effect of LI not only on N1m, but also on later occurring evoked responses. Thus, LI might influence more elaborated processing steps in audition, as well. However, as this is the first study which found this long latency effect of a notch-filtered masker on the processing of a following tone, further examination and replication of this effect is needed.

Within the literature of auditory processing in masking paradigms, it is important to distinguish between simultaneous and sequential masking. In the current study, we used sequential masking paradigm, i.e., there was a delay (500 ms) between the masking sound and the test tone, and found an effect of LI, replicating the effect reported in several other studies on sequential masking [1–4]. In a study using simultaneous masking, i.e., a concurrent presentation of the test tone and the masking sound, however, this effect was reversed [20]: stronger suppression was assessed for a tone when presented within white noise compared to white noise with narrow notches around the frequency of the tone. These diverging results might be explained by the observation that the effect of LI lasts for at least 500 ms up to several seconds [14] and might therefore be longer in duration than direct stimulus-specific adaptation effects which do not rely on LI in the auditory cortices. Hence, the effect of LI might therefore be more pronounced in sequential masking paradigms.

To the best of our knowledge this is the first study directly addressing the question of the influences of focused attention on the effect of LI in the human auditory cortex. We showed that the effect of LI could in fact be influenced by a modulation of the attentional focus: In two time intervals following the N1m time window, the effect of LI was stronger when subjects directed their attention to the auditory modality compared to when they were distracted from the auditory modality. These results indicate that the effect of attention on LI shows its impact later than originally expected. In the time window of the N1m, usually prone to the effect of LI, no significant effect of attentional modulation could be demonstrated, although a hint for a modulatory influence of attention could be observed in descriptive data (cf. Fig 4). This finding fits to the observation, that at longer latencies after stimulus onset, more robust tuning of neural receptive fields in the auditory cortices due to attentional modulation can be seen, especially in interaction with masking stimuli [34] but also under focused attention in general [15].

Analogue to Jääskeläinen and colleagues [24,25], we assume that focused attention leads to a combination of gain increase and sharper frequency tuning of neurons in the auditory cortices. Hence, our findings of an enhanced LI effect under focused attention can be interpreted as a result of an enhanced neural activity in the secondary auditory cortex as well as a sharper frequency tuning, putatively due to an intensified inhibitory system under focused auditory attention [24]. Thus, it can be argued, that attention not only enhances excitatory input but alters the influence of inhibitory interneurons as well. Taken together and considering the results of Pape and coworkers [27], who found diminished long-term effects of LI on the neural processing under distracted listening to notched music in tinnitus patients, there is growing evidence that LI is modulated by focused attention.

Although LI was typically demonstrated in complete absence of *focused* attention i.e., in passive listening paradigms [1,3,4], the present study points towards the assumption, that a minimum of attentional resources should be directed towards the auditory modality for the effect of LI to occur in later processing stages: Under distracted listening, i.e., while performing an active task concerning the visual modality, the effect of the different masker types on the amplitude of the TT seemed reversed (NWN > WN) compared to the active listening condition (NWN < WN). This effect, however, was only found in post-N1m time windows, while the significant main effect for *noise type* points towards a preserved effect of LI in the N1m time window even under distracted listening. Moreover, we are not able to draw inferences about the difference between active and passive listening on the effect of LI, as we chose to only contrast active against distracted listening. We omitted a passive listening condition, as we wanted to keep the experiment in a proper time limit and control for the vigilance of the subjects (i.e., an active task in both conditions). Hence, it remains unclear whether the “optimal” condition to achieve an effect of LI would be passive or active listening. After our demonstration of a general effect of attention, further studies should now address the question about the dose-effect relation in more detail by including an additional passive listening condition. Furthermore, linking our results and the results of Pape et al. 2014, it would be interesting to test, whether our results can be transferred on longer lasting effects of LI, namely the mechanism of inhibition-induced plasticity. If this holds true, these findings, together with the current study, could underline the importance of an attentional listening to enhance the positive effect of notched music (e.g.,[11]) on perceived tinnitus loudness.

The behavioral results revealed high *d*-prime values in both tasks, indicating that the subjects perceived both tasks as relatively easy and were able to direct their attention successfully to the visual or to the auditory modality according to their instruction. Furthermore, higher *d*-prime values were observed in the visual tasks compared to the auditory task, indicating that this task was easier to perform. The visual task served only to distract the attention from the auditory modality and neural responses to the visual stimuli were not analyzed in this study. Hence, an equal level of task difficulty between the two tasks has not been considered as mandatory for the current study. Furthermore, the post-hoc ANCOVA revealed that the differences in the amount of LI under the visual and the auditory task cannot be explained by differences in task difficulty. Hence, we can conclude that the effects obtained in this study can be attributed to the influence of attention on the effect of LI, rather than to differences in task difficulty or cognitive load.

Taken together, our results underline the robustness of the N1m reduction due to LI, as we were able to show its existence in a new paradigm and by means of a different source reconstruction method than the ECD model used before [2,4]. Furthermore, we found hints for a prolonged influence of LI on the auditory processing, as the effect of the masker on the following TT was preserved in an additional time window after the peak of the N1m. Most importantly, we found first evidence for a predicted influence of top-down processes like the focus of attention on the mechanism of LI. Based on our results, it seems likely that attention modulates the magnitude of LI in auditory cortices and that attentional distraction may disrupt the mechanism of LI in the auditory cortex to some extent, especially at later processing stages.

## Supporting Information

S1 Dataset Dataset containing mean neural activity evoked by the test tone in two significant clusters for the main effect *noise type*. Mean neural activity in the significant clusters for the main effect *noise type* (white noise (WN) vs. notched white noise (NWN)) which survived the cluster-based permutation test. The first cluster was found in the predefined sensory region of interest in a time interval (IOI) between 70 and 105 ms, the second cluster in a time interval between 180 and 268 ms. Data is presented separately for auditory task (aud) and the visual task (vis).
(SAV)

S2 Dataset Dataset containing mean neural activity evoked by the test tone in two significant clusters for the interaction effect *noise type x attentional focus* as well as behavioural data. Mean neural activity in the significant clusters for the interaction effect *noise type* (white noise (WN) vs. notched white noise (NWN)) *x attentional focus* (auditory (aud) vs. visual (vis)) which survived the cluster-based permutation test. The first cluster was found in the predefined sensory region of interest in a time interval (IOI) between 147 and 187 ms, the second cluster in a time interval between 295 and 362 ms. Behavioural data: d-prime values, hit-rates and false-alarm rates for both tasks separately and the difference between the two d-prime values.
(SAV)
